# Dynamic Structural Changes and Thermodynamics in Phase Separation Processes of an Intrinsically Disordered–Ordered Protein Model

**DOI:** 10.1002/anie.202112738

**Published:** 2021-12-06

**Authors:** Steffen Lüdeke, Philipp Lohner, Lara G. Stühn, Martin U. Betschart, Matthias C. Huber, Andreas Schreiber, Stefan M. Schiller

**Affiliations:** ^1^ Institut für Pharmazeutische und Biomedizinische Wissenschaften (IPBW) Johannes Gutenberg-Universität Mainz Staudinger Weg 5 55128 Mainz Germany; ^2^ Institut für Pharmazeutische Wissenschaften Albert-Ludwigs-Universität Freiburg Albertstrasse 25 79104 Freiburg Germany; ^3^ Zentrum für Biosystemanalyse (ZBSA) Albert-Ludwigs-Universität Freiburg Habsburgerstrasse 49 79104 Freiburg Germany; ^4^ Cluster of Excellence livMatS @ FIT—Freiburg Center for Interactive Materials and Bioinspired Technologies Albert-Ludwigs-Universität Freiburg Georges-Köhler-Allee 105 79104 Freiburg Germany; ^5^ IMTEK—Institut für Mikrosystemtechnik Albert-Ludwigs-Universität Freiburg Georges-Köhler-Allee 103 79104 Freiburg Germany

**Keywords:** circular dichroism, elastin-like proteins, intrinsically disordered proteins, matrix least-squares global fitting, protein assembly

## Abstract

Elastin‐like proteins (ELPs) are biologically important proteins and models for intrinsically disordered proteins (IDPs) and dynamic structural transitions associated with coacervates and liquid–liquid phase transitions. However, the conformational status below and above coacervation temperature and its role in the phase separation process is still elusive. Employing matrix least‐squares global Boltzmann fitting of the circular dichroism spectra of the ELPs (VPGVG)_20_, (VPGVG)_40_, and (VPGVG)_60_, we found that coacervation occurs sharply when a certain number of repeat units has acquired β‐turn conformation (in our sequence setting a threshold of approx. 20 repeat units). The character of the differential scattering of the coacervate suspensions indicated that this fraction of β‐turn structure is still retained after polypeptide assembly. Such conformational thresholds may also have a role in other protein assembly processes with implications for the design of protein‐based smart materials.

## Graphical abstract

Elastin‐like proteins (ELPs) undergo reversible coacervation above a certain transition temperature. The thermodynamic analysis of temperature‐dependent circular dichroism of ELPs of different chain‐length revealed that this is preceded by a step‐by‐step increase of β‐turn structure. When a certain threshold is reached, phase separation occurs. The conformational ratio in the assembled protein particles still corresponds to this threshold.

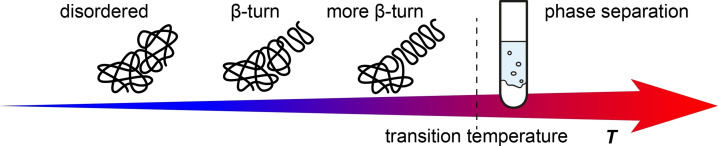

## Introduction

The dynamic transitions between different states of structural order/disorder and phase changes involving intrinsically disordered proteins (IDPs) or intrinsically disordered regions (IDRs)^[1]^ have a fundamental role in cell signaling, cell division, intracellular transport, cell mechanics, protein degradation, post‐transcriptional regulation, cell cycle control, transcription and translation starting from embryonal development to cell differentiation, proliferation and homeostasis.[^1c^, ^1d^] Protein phase separation (PPS), in particular liquid–liquid phase separation (LLPS) of IDPs, is a universal process in biological systems.^[2]^ Recently, it gained great interest in the context of the formation of membraneless organelles and orchestration of functional protein assemblies in vivo and in vitro controlling essential signaling.[^1a^, ^1b^, ^1e^]

Dynamic and reversible protein assembly and disassembly is controlled by pH, temperature, IDP concentration, post‐translational modification, and the binding of specific ligands—parameters also known for elastin‐like protein (ELP) structure and phase transitions.^[1e]^ ELPs are polypeptides of the form (VPGXG)_*n*_, derived from hydrophobic domains of elastin, a fibrous structural protein from the extracellular matrix conferring elasticity to connective tissue of, for example, blood vessels, lung parenchyma, skin, or tendons.^[3]^ ELPs (non‐crosslinked) are prototypic for exhibiting miscible phases at temperatures below a lower critical solution temperature, however, at temperatures above their individual transition temperature (*T*_t_), they form insoluble aggregates,^[4]^ a process called inverse temperature transition (ITT).^[5]^ As this process is fully reversible, ELP‐derived biomaterials are used in responsive hydrogels^[6]^ or temperature‐sensitive therapeutics,^[7]^ as building blocks for the formation of dynamic protein membrane‐based compartments (PMBC),^[8]^ and as minimal cell models.[^8a^, ^9^] Due to these properties, ELPs are excellent models for the understanding of protein folding and assembly[^5a^, ^10^] and they serve as powerful and readily accessible examples for IDP‐dependent processes (see pioneering work by Chilkoti and co‐workers).^[11]^ As IDP‐based molecular systems, they allow for stimulus‐responsive phase‐separated formation of organelle‐like structures.[^8b^, ^9b^, ^10^, ^12^]

However, despite extensive efforts employing NMR,^[13]^ fluorescence techniques,^[14]^ microscopy methods,^[15]^ and cryo‐electron tomography,^[16]^ our knowledge of the molecular processes during PPS, particularly the role of dynamic structurally ordered or disordered protein conformations, right before PPS occurs, remain largely in the dark.^[17]^ Due to their sensitivity towards secondary structure, chiroptical spectroscopic techniques such as circular dichroism (CD) are a promising alternative for the analysis of conformational changes in solution.^[18]^ However, for studying dynamic PPS, structural data from two phases have to be retrieved—a generally challenging task, as spectra might be adulterated by scattering effects.^[19]^ This leaves only a narrow window of experimental conditions in which both phases exist next to each other, namely conditions where suspended particles are small enough to minimize interference of absorbance and scattering, and large enough to allow a reliable analysis of generally small signals. Still, small changes in chiroptical spectra can be highly significant if they coincide with a physical model describing the underlying dynamic process, as recently demonstrated for the global analysis of spectral changes occurring over time,^[20]^ in response to pH,^[21]^ ligand binding,^[22]^ or chemical modification.^[23]^

Herein, we employ this concept introducing a strategy of using CD spectroscopy to diagnose phase transitions. We designed and cloned ELP constructs for three ELPs with different chain lengths, namely (VPGVG)_20_, (VPGVG)_40_, and (VPGVG)_60_, produced them in *E. coli*, and purified them using His‐tag affinity chromatography (Figure S1). We measured CD spectra of the three ELPs in the temperature range from 10 °C to 80 °C in buffer with low salt content (10 mM NaH_2_PO_4_/Na_2_HPO_4_ containing 20 mM of NaCl buffer at pH 7.5). By the use of matrix least‐squares (MLS) global fitting of the temperature‐dependent CD spectra using a Boltzmann‐derived model, we determined the thermodynamic parameters Δ*H* and Δ*S* associated with conformational transitions, and, if applicable, with the assembly to insoluble aggregates. In line with previous findings accessed by NMR techniques,^[24]^ the relative proportion of transient β‐turn structure to disordered structure before ITT obtained from spectral fitting, and the results from analyzing differential scattering after ITT, show that a certain amount of disorder is retained during phase separation.

## Results and Discussion

**Figure 1:**
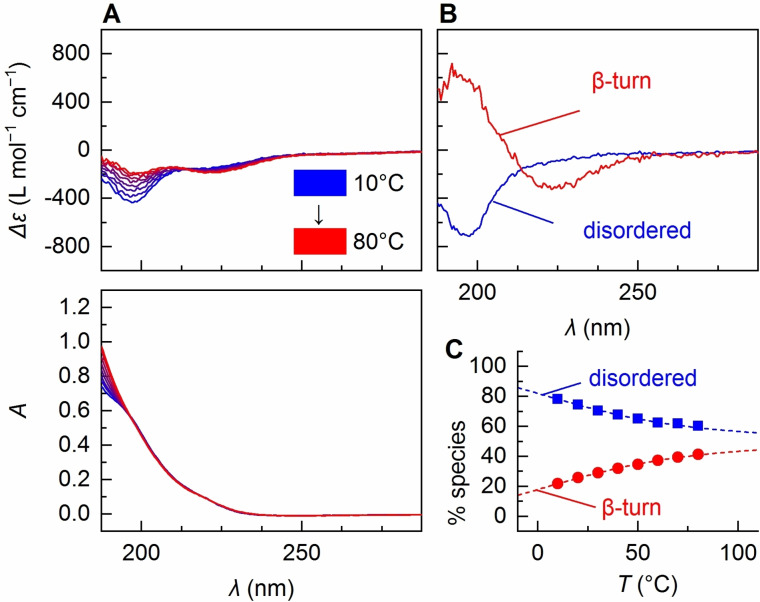
A) Temperature‐dependent CD spectra of (VPGVG)_20_ and the parent absorbance spectra. B) Pure species spectra from MLS global fitting. The low‐temperature species spectrum (blue) clearly corresponds to disordered structure. The shape of the high‐temperature species spectrum agrees with β‐turn structure.^[27]^ C) Temperature‐dependent coefficients for low‐temperature species (blue squares) and high‐temperature species (red dots) from back‐fitting of the spectra in (B) to the spectra in (A). Dotted lines correspond to the theoretical coefficients calculated from Equation (1) using the thermodynamic parameters from global fitting (Table 1).

The CD spectra recorded between 10 °C and 60 °C exhibit considerable temperature‐dependent changes (Figure 1 A). Different from larger oligomers of VPGVG (*n=*200) showing a *T*_t_ of 20 °C,^[25]^ at low salt concentrations as used in this study, (VPGVG)_20_ is soluble over the whole observed temperature range, indicated by dynamic light scattering (DLS, Figure S2A) and the fact that pronounced band shape and intensity effects from scattering or absorption flattening are absent in the parent absorbance spectra (Figure 1 A). Therefore, we conclude the CD changes to have their origin in a temperature‐dependent secondary structure transition. The negative intensity at ≈195 nm indicating disordered structure^[18a]^ is clearly reduced at higher temperature accompanied by the increase of a negative intensity at 223 nm. The overlaid spectra display an isodichroic point at 213 nm thus suggesting an interconversion between two temperature‐dependent species.

For fully reversible processes, we can model the transition between different species as a Boltzmann distribution [Eq. 1]:

(1) $$p_{i}=\frac{e^{-\frac{\Delta G_{i}}{kT}}}{\sum_{j=1}^{M}e^{-\frac{\Delta G_{j}}{kT}}}$$

Here, *p_i_* is the proportion of species *i* out of a total number of *M* species, Δ*G_i_* is the Gibbs free energy of species *i* relative to the lowest‐lying species, *k* is the Boltzmann constant and *T* is the temperature. Provided that the entropy *S* of the different states is also different, according to Δ*G*=Δ*H*−*T*Δ*S*, the relative Gibbs free energy itself is also highly temperature dependent. As for ELPs the phase transition is an entropy‐driven process,[^5a^, ^26^] temperature‐dependent changes in the population of a conformational species should be mainly due to a rearrangement of the free energy states: if Δ*G_i_* decreases at increasing *T*, the Boltzmann distribution with respect to higher‐temperature Δ*G* will also deliver a higher proportion of species *i*.

Assuming two states (*M*=2) for (VPGVG)_20_, we fitted the spectra in Figure 1 A using the MLS global fitting method.^[28]^ As a model, we used Equation (S1), which weights spectral amplitudes with *p_i_*, obtained from Equation (1) with respect to the open parameters Δ*H_i_* and Δ*S_i_* (both treated as temperature‐independent). The determination of both parameters in a single experiment is a marked departure from other approaches usually involving a separate determination of Δ*H* from calorimetric measurements. As MLS global fitting takes into account the information of the full spectra, the fitting stability is increased, thereby allowing to resolve both Δ*H_i_* and Δ*S_i_* simultaneously. Furthermore, MLS global fitting delivers the spectrum of each species that contributes to the observed spectra (Figure 1 B; Equations (S2) and (S3)), and, from back‐fitting of these spectra to the observed spectra, the linear coefficients (correspond to the values of *p_i_*) for each species (Figure 1 C). The back‐fitted coefficients highly agree with the theoretical distribution indicating the applicability of the two‐state Boltzmann model. This is also confirmed by small residuals after MLS global fitting (*R=*0.09; Equation (S6), Table S2, Figure S5).

The fitted low‐temperature species spectrum (Figure 1 B) exhibits a strong negative band at 195 nm and a negative shoulder at 220 nm indicating disordered structure.^[18a]^ The high‐temperature species spectrum has a negative band at 222 nm and a positive band at 195 nm being typical for β‐turn structure.^[27]^ This is in agreement with previous observations of β‐turn in ELPs of the (VPGVG)_*n*_ type.^[29]^ The enthalpy difference Δ*H*=5.4 kcal mol^−1^ (0.3 kcal mol^−1^ per repeat unit, Table 1) between the two species is in the range of what is expected for single‐bond rotations in the protein backbone.^[30]^ As the entropy of the system clearly increases (Δ*S*=18 cal mol^−1^, Table 1), the observed transition can be explained by a hydrophobic collapse driven by the entropy gain from the release of water molecules acquiring a tighter packing upon formation of multiple β‐turns.[^5b^, ^31^] According to MLS global fitting of the CD data, in an aqueous solution at 10 °C, (VPGVG)_20_ has already ≈20 % β‐turn structure converging to a 50:50 ratio above 70 °C.

**Table 1:** Thermodynamic parameters of (VPGVG)_20_, (VPGVG)_40_, and (VPGVG)_60_ from MLS global fitting (per repeat unit values given in parentheses).

	Δ*H*_1_ [kcal mol^−1^]	Δ*S*_1_ [cal mol^−1^ K^−1^]	Δ*H*_2_ [kcal mol^−1^]	Δ*S*_2_ [cal mol^−1^ K^−1^]
(VPGVG)_20_	5.4 (0.3)	18 (0.9)	–	–
(VPGVG)_40_	9.9 (0.2)	33 (0.8)	89 (2.2)	271 (6.8)
(VPGVG)_60_	11.2 (0.2)	36 (0.6)	158 (2.6)	345 (5.8)

**Figure 2:**
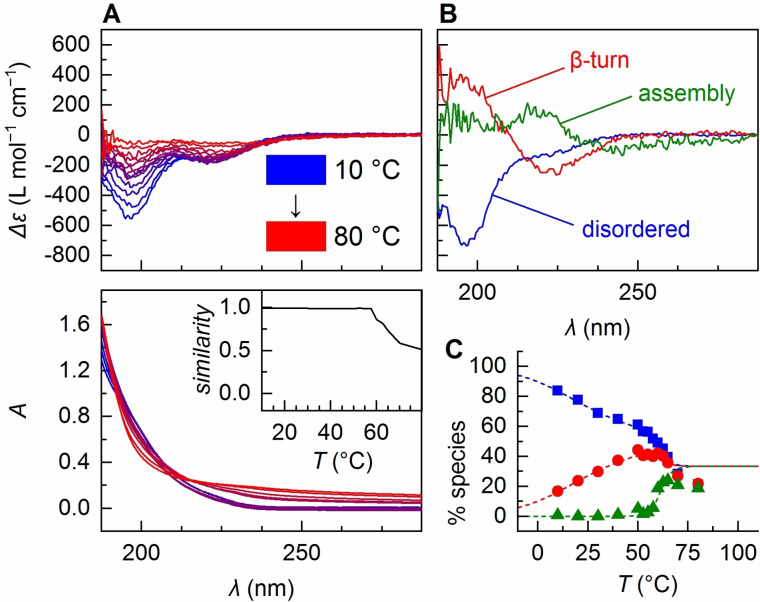
A) Temperature‐dependent CD spectra of (VPGVG)_40_ and the parent absorbance spectra. Inset: cosine similarity of absorbance spectra and the average absorbance of (VPGVG)_20_. B) Pure species spectra from MLS global fitting. The spectrum labeled as “assembly” also contains negative features from the disordered and β‐turn spectra, which accounts for intensity loss mainly due to precipitation. C) Temperature‐dependent coefficients from back‐fitting (disordered: blue squares, β‐turn: red dots, assembly: green triangles). Before ITT, the coefficients agree excellently with the model (dotted lines, see Table 1 for parameters) but are lower than expected above phase transition.

In the case of (VPGVG)_40_, the situation is more complex. Even at 10 °C, in addition to the strong negative band at 195 nm, the CD spectra exhibit a clearly visible negative band at 222 nm already indicating a considerable amount of β‐turn. Above ≈60 °C, which is around the *T*_t_ previously found for similar ELPs,^[5a]^ the spectra become less intense (Figure 2 A), concomitant with an obvious change in shape and intensity of the parent absorbance spectra (Figure 2 A). These changes can be explained by scattering and absorption flattening,[^19b^, ^32^] or even precipitation of particles above 60 °C.

For a quantitative assessment, we assumed three states, disordered, β‐turn, and a third species with the working title “assembly”. Different from solution state spectra, the fitting of the spectral changes associated with this third component is considerably more challenging, as considerations made for the fitting of solution spectra might not apply for suspensions. Furthermore, slow equilibration due to the phase transition might lead to transient accumulation of the solid species at the expense of the two solution phase species, while Equation (1) suggests a relative ratio of 1:1:1 at infinite temperature. To minimize errors from the abovementioned sources, we modified the fitting by applying the highest weight on spectra containing least scattering contributions. In addition, a similarity criterion with respect to reference spectra for disordered and β‐turn structure (Figure 1 B) was included in the minimization function (maximum a posteriori estimation, see SI for details).

**Figure 3:**
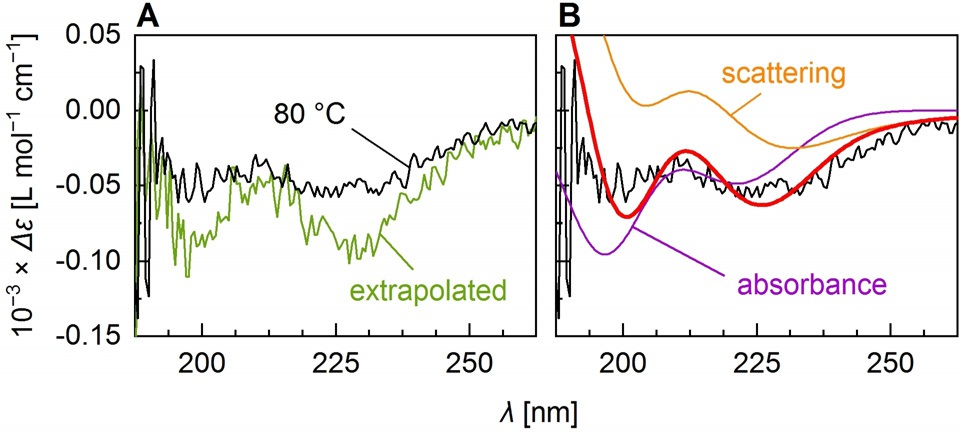
A) Spectrum measured for (VPGVG)_40_ at 80 °C and the spectrum extrapolated to high temperatures from MLS global fitting. B) Fit (red line) of the pure CD spectra and differential scattering spectra to the 80 °C CD spectrum. The purple spectrum (absorbance) is the spectrum obtained from a linear combination of the difference absorbance spectra, the orange spectrum (scattering) is the linear combination of the two differential scattering spectra, for each using the coefficients from the fit.

The pure spectra corresponding to species 1 and species 2 clearly show the spectral features expected for disordered and β‐turn structure (Figure 2 B). The interconversion between these two species is associated with Δ*H*_1_=9.9 kcal mol^−1^ and Δ*S*_1_=33 cal mol^−1^ K^−1^ (Table 1). For the third species (“assembly”), the fit delivers a spectrum containing negative features of disordered and β‐turn. It arises sharply at ITT conditions (≈60 °C) with Δ*H*_2_=89.1 kcal mol^−1^. This high transition enthalpy is compensated by an entropy gain of Δ*S*_2_=270 cal mol^−1^ K^−1^ leading to the observed sharp transition (Table 1, Figure 2 C). The per repeat unit energy intake upon phase transition is 2.2 kcal mol^−1^, which is twice the transition enthalpy of 1.1 kcal mol^−1^ previously observed for variable‐chain‐length (VPGVG)_*n*_ by dynamic scanning calorimetry.^[33]^ The per repeat entropy change of 6.8 cal mol^−1^ K^−1^ is in the range of transition entropy changes obtained previously from calorimetry‐derived heats of transition and the *T*_t_ of different ELPs.[^5a^, ^34^] The spectrum recorded at 80 °C closely resembles a spectrum one would obtain from extrapolating the results from global fitting to infinite temperature (Figure 3 A), albeit with lower signal intensities, presumably due to precipitation of aggregated material (also reflected by the deviation of back‐fitted proportions from the model in Figure 2 C and an *R*‐value of 0.26; Table S2, Figure S6). Nevertheless, this qualitative similarity confirms that the model (with modified fitting parameters) predicts the temperature‐dependent CD changes correctly and indicates that the transition is more or less completed at 80 °C.

Both the extrapolated and the 80 °C spectra show clear features of disordered and β‐turn structure in solution, as well as features that suggest the presence of a third component. Particularly the broadening of the negative band at 224 nm towards longer wavelengths cannot be explained by a combination of the solution structure spectra. Dynamic light scattering (DLS) experiments of (VPGVG)_40_ indicate the formation of particles with diameters distributed around 164 nm (Figure S2 B), which is in the range of what has been determined previously for (VPGVG)_40_ particles.^[35]^ As the size of these particles is about the wavelength of the circularly polarized light used in the CD experiment, the above‐mentioned band broadening likely has its origin in differential scattering caused by the structure of the newly formed particles.^[19b]^

The thermodynamic model and the temperature‐dependent coefficients obtained for (VPGVG)_40_ (Figure 2 C) suggest that PPS sharply occurs at the point where disordered and β‐turn structure exist in a ≈1:1 ratio. We wondered whether this ratio may also have consequences on the conformational composition of aggregates formed from these two species above ITT.

It is generally difficult to tell apart contributions from the difference absorbance, i. e. “true CD”, of solution species from those that are present in particles. Differential scattering, on the other hand, exclusively occurs above a certain radius of gyration. Therefore, in the following we will focus our attempts of assessing the structural composition of the aggregates on the interpretation of the scattering portion of the observed CD above ITT. While differential scattering is generally disesteemed as a source of spectral distortions, depending on size and wavelength, it can make a significant contribution to an apparent CD spectrum and also contains a lot of structural information, as demonstrated previously for the identification of micrometer‐scale helix structure.^[36]^

In the case of (VPGVG)_40_, where the diameter of the particles formed above ITT is in the few hundred nanometer range (see above), the contribution from differential scattering Δ*s* to the CD spectrum of a suspension is a function of *∂n*(*λ*)/∂*c*, the gradient of the refractive index *n* of the solution with respect to the concentration *c* of the scattering particle, and *∂*Δ*n*(*λ*)/∂*c*, the difference refractive index gradient for left minus right circularly polarized light (equation (S4)).[^19b^, ^37^] The latter is equivalent to the optical rotatory dispersion (ORD) spectrum, which explains the previously observed resemblance of distortions in the CD of suspensions to an ORD spectrum.[^19d^, ^38^] These wavelength‐dependent gradients can be calculated from the Kramers–Kronig transform (KKT) of the molar absorptivity spectrum *ϵ*(*λ*) and the molar difference absorptivity spectrum Δ*ϵ*(*λ*) (Equation (S5)), thereby allowing the simulation of differential scattering spectra of disordered and β‐turn structure (see SI and Figures S3 and S4 for details).

To estimate the relative proportion of disordered and β‐turn structure in the particles, we fitted the difference absorbance and the simulated differential scattering spectra of disordered and β‐turn structure (Figure S3) to the spectrum recorded at 80 °C (Figure 3 B). Inclusion of the CD difference absorbance was necessary, as the 80 °C spectrum still contains a considerable amount of “true CD” of the particles and the surrounding solution. With respect to the difference absorbance portion (purple spectrum in Figure 3 B) we obtained a ratio disordered:β‐turn of 1.8:1.0. Interestingly, the differential scattering portion alone (orange spectrum in Figure 3 B) is well described by a disordered:β‐turn ratio of the simulated differential scattering spectra of 1.0:1.0. This actually agrees with the hypothesis from above suggesting that disordered and β‐turn structure are present in a stoichiometric ratio of ≈1:1 in particles formed from the assembly of (VPGVG)_40_. Still, at this point it is not clear how exactly this ratio describes the distribution of secondary structure in the assembled particle. It could either mean that about one half of the ELP molecules exists as disordered structure, and the other half as β‐turns, or that about one half of the repeat units of each ELP molecule, in the case of (VPGVG)_40_ ≈20 units, acquires β‐turn structure while the other half remains disordered. In the latter case, it is also possible that the number of ≈20 units as β‐turn structure denotes a threshold above which PPS occurs. If this was the case, the 1:1 ratio would be uniquely observed for (VPGVG)_40_ and ELPs of other chain length should deliver different results.

**Figure 4:**
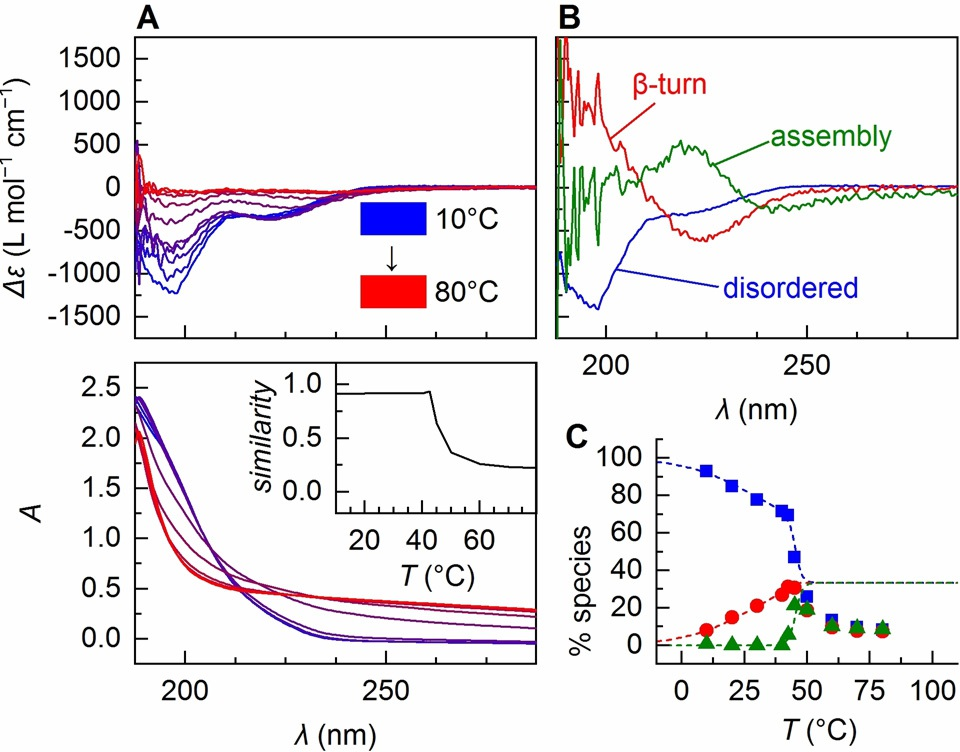
A) Temperature‐dependent CD spectra of (VPGVG)_60_ and the parent absorbance spectra. Inset: cosine similarity of absorbance spectra and the average absorbance of (VPGVG)_20_. B) Pure species spectra from MLS global fitting. C) Temperature‐dependent coefficients from back‐fitting (disordered: blue squares, β‐turn: red dots, assembly: green triangles). Dotted lines are the theoretical values according to the model (see Table 1 for parameters).

To assess the problem whether phase separation depends on the conformational composition of the solution ensemble or tertiary structure of individual chains, we also performed a CD analysis of a third ELP, namely (VPGVG)_60_, in the same temperature range. Here, a strong deformation of the absorbance spectra and a considerable decrease in CD intensity are observed above 42.5 °C (Figure 4 A). In contrast to (VPGVG)_40_, where most of the interconversion between disordered and β‐turn occurs well separated from the steep phase transition above 60 °C, for (VPGVG)_60_ a third species already appears at ≈40 °C (Figure 4 C). DLS experiments suggest formation of particles of ca. 250 nm size at 60 °C, which is similar to (VPGVG)_40_, however, with a considerably broader distribution of diameters (Figure S2 C).

**Figure 5:**
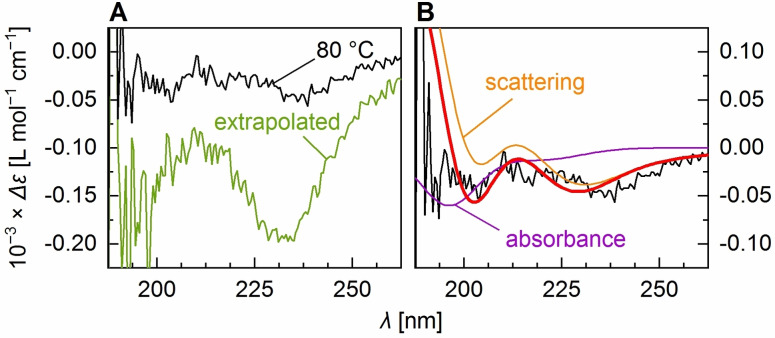
A) Spectrum measured for (VPGVG)_60_ at 80 °C and the spectrum extrapolated to high temperatures from MLS global fitting. B) Fit (red line) of the pure CD spectra and constructed differential scattering spectra to the 80 °C CD spectrum. Purple spectrum: absorbance contribution; orange spectrum: scattering contribution.

The Boltzmann fitting of the CD data (Figure 4 B,C) delivers Δ*H*_1_=11.2 kcal mol^−1^ and Δ*S*_1_=36 cal mol^−1^ K^−1^ (Table 1, *R*=0.35; Table S2, Figure S7). Interestingly, here the assembly occurs nearly concomitantly with the interconversion to β‐turn with Δ*H*_2_=158 kcal mol^−1^ and Δ*S*_2_=345 cal mol^−1^ K^−1^ (Table 1). In contrast to (VPGVG)_40_, ITT even starts at β‐turn proportions of ≈30 % (Figure 4 C), suggesting a stoichiometric ratio of 2:1 instead of 1:1. The analysis of the 80 °C spectrum (Figure 5 A) arrives at a similar conclusion. The fitting of the differential scattering spectra constructed from the refractive index gradient spectra (Figure S4) delivered a relative ratio disordered:β‐turn of 1.8:1 in the scattering regime (Figure 5 B). Even though the agreement between the observed and the simulated spectrum is lower than for (VPGVG)_40_, the qualitative result remains that in assemblies of (VPGVG)_60_ only about a third of the conformational composition is β‐turn structure. This agrees with the second scenario discussed above, namely, that coacervation occurs when a certain threshold of repeat units in β‐turn conformation (for this ELP ≈20 units) is reached, and is in accordance with previous findings from NMR studies that proline‐rich motifs nucleate as low populations (≈30 %) of an unstable intramolecular β‐turn structure in the hydrophobic domains.^[24]^

## Conclusion

Different from other proteins, where the formation of β‐structure is rather an attribute of than a prerequisite for PPS,^[39]^ our analysis revealed a transition between disordered and β‐turn conformation that is clearly preceding ITT in (VPGVG)_40_ and (VPGVG)_60_. Interestingly, at the given conditions, ITT occurs at ≈50 % β‐turn for (VPGVG)_40_ and ≈30 % for (VPGVG)_60_. Fitting the differential scattering portion in suspension CD spectra above ITT suggests that these proportions of β‐turn are also retained in particles formed from (VPGVG)_40_ and (VPGVG)_60_. This leads to the conclusion that, irrespective of chain length, a certain threshold of ≈20 repeat units in β‐turn conformation has to be reached for PPS to occur, thereby possibly engendering a reduction of the radius of gyration of each monomer, which coincides with a collapse transition,^[4]^ while the remaining portion of the molecule remains disordered.^[24]^ Despite the fact that different ELPs exhibit different ITT depending on sequence, concentration, presence of salts, or other parameters, we hypothesize that for each of these settings a certain β‐turn threshold for PPS may exist. Under the chosen experimental conditions, (VPGVG)_20_ is simply too short to reach this threshold, so it will never undergo an ITT, no matter how high the temperature will be.

We expect the findings about this phenomenon and its thermodynamic implications to have far‐reaching consequences for the general understanding of IDP‐based stimulus‐responsive phase separation processes in vivo and in vitro, thereby facilitating the design of temperature‐controlled “smart” materials.

## Conflict of interest

The authors declare no conflict of interest.

## Supporting information

As a service to our authors and readers, this journal provides supporting information supplied by the authors. Such materials are peer reviewed and may be re‐organized for online delivery, but are not copy‐edited or typeset. Technical support issues arising from supporting information (other than missing files) should be addressed to the authors.

Supporting Information
