# Supplementary material for: Dynamic Structural Changes and Thermodynamics in Phase Separation Processes of an Intrinsically Disordered–Ordered Protein Model
Source: Angew Chem Int Ed Engl. 2021 Dec 6;61(3):e202112738. doi: 10.1002/anie.202112738 (PMC9299898; doi:10.1002/anie.202112738)
Supplement: Supplementary file 1 — Supporting Information [file ANIE-61-0-s001.pdf]

Angewandte Chemie
Eine Zeitschrift der Gesellschaft Deutscher Chemiker

Supporting Information

Dynamic Structural Changes and Thermodynamics in Phase Separation Processes of an Intrinsically Disordered–Ordered Protein Model

Steffen Lüdeke, Philipp Lohner, Lara G. Stühn, Martin U. Betschart, Matthias C. Huber, Andreas Schreiber, and Stefan M. Schiller**

anie_202112738_sm_miscellaneous_information.pdf

Contents

I. Preparation of elastin-like proteins (ELP) S2

Protein sequences S3

II. Fitting of CD spectra S4

Data weighting S4

Fitting model and back calculation of the pure spectra S5

Confidence of matrix least-squares (MLS) global fitting S9

III. Supplementary references S11

Materials and Methods

Preparation of elastin-like proteins (ELP)

Design and cloning of the ELP constructs

For the generation of $(VPGVG)_{20}$, $(VPGVG)_{40}$, and $(VPGVG)_{60}$ the “one-vector-toolbox-platform” (OVTP) was applied as modular cloning approach. OVTP was used for cloning of the highly repetitive sequences of the ELP constructs and their constituents starting with the assembly of synthetic oligonucleotides. OVTP provides a basic plasmid backbone with special restriction sites where individual DNA blocks can be incorporated, replaced or substituted and directly used as an *E. coli* expression vector. DNA blocks for different ELPs were excised with SacI and EarI from a library of existing sequence blocks and assembled into the plasmid backbone as described in Huber et. al.[12c] Correct assembly was confirmed by sequencing (GATC GmbH). The final plasmids used for protein expression were named pET28 NMBxL-His-V20, pET28 NMBxL-His-V40 pET28 NMBxL-His-V60.

Polypeptide production and purification

E. coli strains *BL21 (DE3)* were used for the expression of the desired protein constructs. Bacteria were grown while shaking at 250 rpm in LB medium at 37°C until they reached an OD_{600} of 0.7. At this point, expression of the ELP-coding gene was induced with 1 mM IPTG (final concentration). After shaking for 6–7 h at 20°C and 180 rpm cells were harvested by centrifugation at 4,000 *g* for 30 min. After resuspension of the bacteria in lysis buffer (50 mM Tris-HCl pH 8.0, 500 mM NaCl, 20 mM imidazole), 1 mM TCEP (final concentration) and 10 µg mL^{-1} lysozyme per liter of culture volume were added and incubated on ice for 30 min. Cell lysis was induced by freezing the cells in liquid nitrogen twice followed by sonication and centrifugation of the lysate at 10,000 *g* for 40 min. Finally, the supernatant was loaded onto a Ni-NTA-column (*Macherey-Nagel*), washed and eluted with 50 mM Tris pH 7.5, 500 mM NaCl, and 50 mM-300 mM imidazole. For purity analysis and identification a standard 10% Tris/Glycine SDS-PAGE was applied (Figure S1).

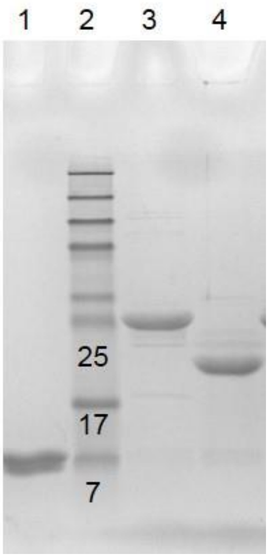

Figure S1. SDS Page of histag purified ELP. Depicted are in lane 1 His-V20 (9.9 kDA), in lane 2 protein ladder standard with indicated kDa, in lane 3 His-V40 (18.1 kDa) and in lane 4 His-V60 (20.1kDa).

Determination of the ELP concentration

After purification, ELPs were dialyzed against milliQ water using a 12–14 kDa MWCO cellulose mixed ester membrane (Roth). After dialysis, the ELP solution was shock frozen in liquid nitrogen and placed in a pre-cooled ALPHA 2-4 LSC freeze-dryer (Christ, Osterode, Germany). The ELP solution was freeze-dried at −20°C to −80°C and 10^{-6} mbar for 48 h. The polypeptide dry weight was used to determine the amount of the sample. After weighing, the lyophilized polypeptide sample was resuspended in 10 mM NaH_2PO_4/ Na_2HPO_4 buffer containing 20 mM of NaCl buffer (pH 7.5) at a final concentration of 1 mg mL^{-1} (stock solution for all further analyses).

Table S1. ELP properties calculated with Expasy[40] and the concentration used for CD measurements in 10 mM NaH_2PO_4/Na_2HPO_4, 20 mM NaCl, pH 7.5.

ELP construct	aa number	MW (Da)	pI (calculated)	*c* (mg mL^{-1})	*c* (µmol L^{-1})
His-$(VPGVG)_{20}$	115	9880.50	6.56	1.00	101
His-$(VPGVG)_{40}$	215	18070.20	6.56	1.00	55
His-$(VPGVG)_{60}$	315	26259.90	6.56	1.00	38

Protein sequences

His-$(VPGVG)_{20}$

MDPMSSSGHHHHHHGVPGVGVPGVGVPGVGVPGVGVPGVGVPGVGVPGVGVPGVGVPGVGVPG
VGVPGVGVPGVGVPGVGVPGVGVPGVGVPGVGVPGVGVPGVGVPGVGVPGVG

His-$(VPGVG)_{40}$

MDPMSSSGHHHHHHGVPGVGVPGVGVPGVGVPGVGVPGVGVPGVGVPGVGVPGVGVPGVGVPG
VGVPGVGVPGVGVPGVGVPGVGVPGVGVPGVGVPGVGVPGVGVPGVGVPGVGVPGVGVPGVGV
PGVGVPGVGVPGVGVPGVGVPGVGVPGVGVPGVGVPGVGVPGVGVPGVGVPGVGVPGVGVPGV
GVPGVGVPGVGVPGVGVPGVGVPGVG

His-$(VPGVG)_{60}$

MDPMSSSGHHHHHHGVPGVGVPGVGVPGVGVPGVGVPGVGVPGVGVPGVGVPGVGVPGVGVPG
VGVPGVGVPGVGVPGVGVPGVGVPGVGVPGVGVPGVGVPGVGVPGVGVPGVGVPGVGVPGVGV
PGVGVPGVGVPGVGVPGVGVPGVGVPGVGVPGVGVPGVGVPGVGVPGVGVPGVGVPGVGVPGV
GVPGVGVPGVGVPGVGVPGVGVPGVGVPGVGVPGVGVPGVGVPGVGVPGVGVPGVGVPGVGVP
GVGVPGVGVPGVGVPGVGVPGVGVPGVGVPGVGVPGVGVPGVGVPGVGVPGVGVPGVGVPGVG

CD spectroscopy

CD and UV/visible absorbance spectra in the spectral range of 180−350 nm were recorded on a J-810 Spectropolarimeter (Jasco, Tokyo, Japan), equipped with a Model PFD- 425S Peltier element (Jasco) at different temperatures between 10 and 80 °C. ELP solutions (9.7 µM) in deionized water were placed in a 1 mm quartz cuvette (Hellma, Müllheim, Germany). The spectra were measured using a scanning speed of 50 nm min^{-1}, a step size of 0.5 nm, a bandwidth of 1 nm, a response time of 1 s, and an accumulation of 3 scans. The spectra were background-corrected by subtracting water spectra recorded at the corresponding temperatures.

Analysis of the CD data

Matrix least-squares (MLS) global fitting

The MLS analysis was performed in MATLAB (MathWorks, Natick, MA, USA) in a similar way as described previously for the analysis of other dynamic processes.[20–23, 41] In brief, a matrix **A** containing the temperature-dependent spectra was subject to singular value decomposition (SVD) delivering three matrices **U**, **S** and **V** according to the decomposition $\mathbf{A} = \mathbf{U} \times \mathbf{S} \times \mathbf{V}^T$, where the columns of **U** contain the orthornormal spectra, i. e. spectra containing the spectral contributions that are correlated throughout the whole data set, the columns of **V** contain the associated temperature-dependent coefficients, and **S** is a diagonal matrix with the singular values.[42] Assuming that the activation energy between the conformational states is low,[43] we neglected kinetic effects in spectral fitting. The open parameters ΔH and ΔS were thus obtained from non-linear least-squares fitting of a Boltzmann model (see Results and Discussion) to the coefficients in **V**. The fitted parameters delivered the partition between the two states for $(VPGVG)_{20}$ and the partition between three states for $(VPGVG)_{40}$ and $(VPGVG)_{60}$, respectively, which allowed obtaining the associated amplitude spectra and pure species spectra after matrix inversion. While $(VPGVG)_{20}$ was fitted using least-squares as the only convergence criterion, for the fitting of $(VPGVG)_{40}$ and $(VPGVG)_{60}$ an approach similar to the maximum *a posteori* method was employed. Here, the similarity of the first two pure spectra, which are expected to correspond to disordered and β-turn structure, to the pure spectra obtained for $(VPGVG)_{20}$ was included as a second convergence criterion (noise-free spectra from Gaussian fitting served as reference spectra). As a measure for spectral similarity, we used cosine similarity ($\cos\theta$), which, independently from relative size of the spectra, delivers similarity values between 1 (identical) and 0 (dissimilar):

$$\cos\theta = \frac{\sum_{\lambda_i} CD_{\lambda_i,\mathrm{pure}} CD_{\lambda_i,\mathrm{ref}}}{\sqrt{\sum_{\lambda_i} CD^2_{\lambda_i,\mathrm{pure}}}\sqrt{\sum_{\lambda_i} CD^2_{\lambda_i,\mathrm{ref}}}}$$

In the minimization function, the similarity term and the least-squares term are multiplied by the regularization parameters λ_{sim} and λ_{lsq}, respectively. As temperature-dependent weighting led to different amounts of spectral information in $(VPGVG)_{40}$ compared to $(VPGVG)_{60}$, also different regularization parameters have been applied: $\lambda_{sim} = 10^4$ and $\lambda_{lsq} = 10^5$ for $(VPGVG)_{40}$; $\lambda_{sim} = 10^5$ and $\lambda_{lsq} = 10^5$ for $(VPGVG)_{60}$.

Data weighting

As the quality of the data strongly varied with wavelength and temperature, the data in **A** was weighted by wavelength (for example, intensities at <185 nm were ignored) and temperature-dependent functions in the SVD and the following fitting steps. Noise functions for $(VPGVG)_{20}$, $(VPGVG)_{40}$, and $(VPGVG)_{60}$ were obtained from Fourier-filtering of each temperature-dependent CD spectrum with a 4 nm cut-off and taking the average over all spectra.

To account for temperature-dependent uncertainty of the CD data (for example spectra recorded above ITT are considerably smaller and therefore less reliable), in singular value decomposition (SVD) and matrix least-squares (MLS) global fitting, the CD data were weighted by temperature-dependent uncertainty functions. Temperature-dependent noise-levels were obtained from averaging the results from Fourier-filtering (see above) over all wavelengths. To obtain the uncertainty functions, the temperature-dependent noise levels were additionally weighted by a similarity function: in the case of $(VPGVG)_{20}$ the relative deviation from the average absorbance spectrum; in the case of $(VPGVG)_{40}$ and $(VPGVG)_{60}$ the cosine similarity of each absorbance spectrum and the average absorbance spectrum of $(VPGVG)_{20}$ (a spectrum considered as scattering-free).

While the first criterion weights the most reliable data points, the second favors a fitting with a structurally meaningful result.

Fitting model and back calculation of the pure spectra

Each temperature-dependent CD spectrum is modeled as the Boltzmann-weighted sum of M amplitude spectra (a_0, a_1,…a_M) for M considered equilibrium species:

$$CD(T) = \sum_{j=0}^{M-1} a_j p_j(T) \qquad \text{(S1)}$$

Here, $p_j(T)$is the Boltzmann distribution as given in equation (1) in the main text. The amplitude spectra are obtained from:[42]

$$\mathbf{D} = \mathbf{A}\mathbf{P}^{\mathbf{T}+}, \qquad \text{(S2)}$$

where **D** is a matrix whose columns correspond to the amplitude spectra a_0, a_1,…a_M, **A** is the matrix containing the temperature-dependent CD spectra, and $\mathbf{P}^{\mathbf{T+}}$ is the pseudoinverse of the transpose of matrix **P**. The columns of **P** contain the Boltzmann distributions p_0, p_1,…p_M, each calculated with the corresponding ΔH and ΔS from non-linear least-squares fitting of the Boltzmann model to the temperature-dependent SVD coefficients.

The pure species spectra b_0, b_1,…b_M, shown in Figures 2, 3, and 5 (main text) are back calculated from (j = 0, 1, …, M−1):

$$b_j = (j+1)a_j - j a_{j-1} \qquad \text{(S3)}$$

Simulation and fitting of differential scattering spectra

For the fitting of putative contributions from differential scattering, we simulated differential scattering spectra from the pure species spectra of disordered and β-turn structure obtained from MLS global fitting of $(VPGVG)_{40}$ and $(VPGVG)_{60}$ and a representative absorbance spectrum. The contribution from differential scattering Δ*s* to the CD spectrum of a suspension as derived from statistical fluctuation theory is given by:[44]

$$\Delta s(\lambda) \propto \frac{2V\big(n_0(\lambda)\big)^2}{\lambda^4 \bar{c}} \cdot \frac{\partial n(\lambda)}{\partial c} \cdot \frac{\partial \Delta n(\lambda)}{\partial c} \langle (\partial c)^2 \rangle \qquad \text{(S4)}$$

Here, V is the volume element where the scattering occurs, $n_0(\lambda)$ is the refractive index spectrum of the solvent, λ is the wavelength, $\bar{c}$ is the average concentration in the volume

element, $\partial n(\lambda)/\partial c$ is the gradient of the refractive index n of the solution with respect to the concentration c of the scattering, and $\partial \Delta n(\lambda)/\partial c$ is the difference refractive index gradient for left minus right circularly polarized light. The scattering intensity also depends on $\langle(\partial c)^2\rangle$, the mean quadratic fluctuation in a volume V. It is proportional to $\bar{c}$ and $(1-q)$, where q is the probability of a light beam to meet a particle in a volume element V.

The wavelength-dependent refractive index components $\partial n(\lambda)/\partial c$ and $\partial \Delta n(\lambda)/\partial c$ (in molar absorptivity units) according to

$$\frac{\partial n(\lambda)}{\partial c} = \frac{\ln(10)\lambda}{4\pi}\mathrm{KKT}\big(\varepsilon(\lambda)\big)$$

$$\frac{\partial \Delta n(\lambda)}{\partial c} = \frac{\ln(10)\lambda}{4\pi}\mathrm{KKT}\big(\Delta\varepsilon(\lambda)\big) \qquad \text{(S5)}$$

were calculated after Kramers–Kronig transform (KKT) of the Gauss-fitted absorptive spectra using the KKT-tool by Lucarini et al. in MATLAB.[45] The fitting of pure CD spectra and the differential scattering spectra to the 80 °C spectra of $(VPGVG)_{40}$ and $(VPGVG)_{60}$ was also performed in MATLAB.

Dynamic light scattering (DLS)

Light scattering of solutions of $(VPGVG)_{20}$, $(VPGVG)_{40}$, and $(VPGVG)_{60}$ was measured at 20 °C on a Zetasizer ZS instrument (Malvern Panalytic, Malvern, UK) at a measurement position set at 1.05 mm and a scattering angle of 173°. For optimal conditions for the determination of particle size distributions, the laser transmission was set to automatic attenuation.

Supplementary data

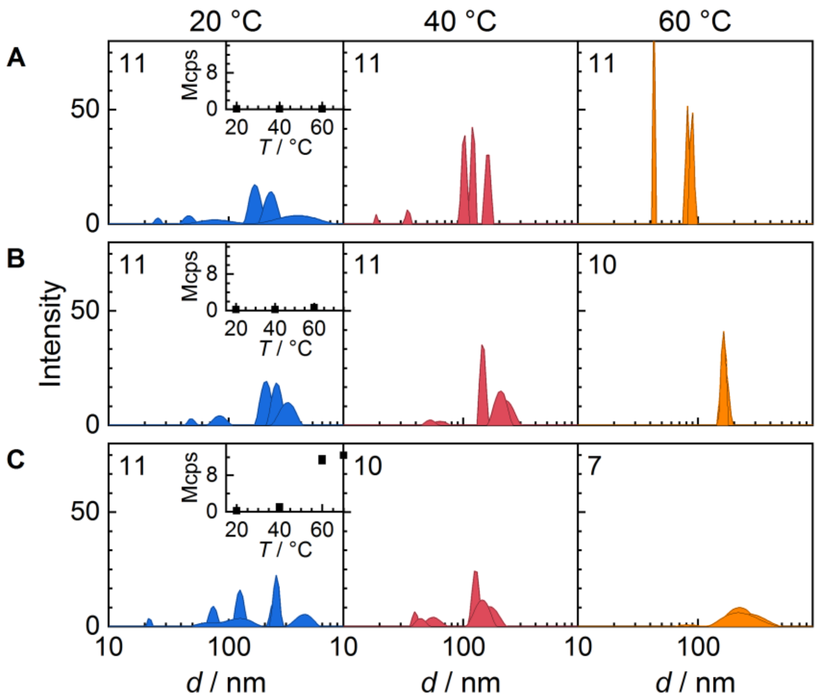

Figure S2. DLS of aqueous solutions of $(VPGVG)_{20}$ (A), $(VPGVG)_{40}$ (B), and $(VPGVG)_{60}$ (C) at 20, 40, and 60 °C. Insets show the count rates (Mcps) for the three species at the three temperatures. Settings of the laser attenuator (7, 10, 11) are shown in the upper left corners. A: The DLS for $(VPGVG)_{20}$ is generally low at all three temperatures (attenuator setting: 11). Therefore, the sharp distributions cannot be assigned to putative ELP assembly. B: DLS increases moderately for $(VPGVG)_{40}$ requiring lower attenuation at 60 °C (10). The DLS intensities at 60 °C are distributed around a diameter of 164 nm. C: For $(VPGVG)_{60}$, DLS increases significantly with temperature requiring attenuator settings of 10 and 7 at 40 and 60 °C, respectively. The DLS intensities at 60 °C are broadly distributed around a diameter of 250 nm ranging from 120 to 450 nm.

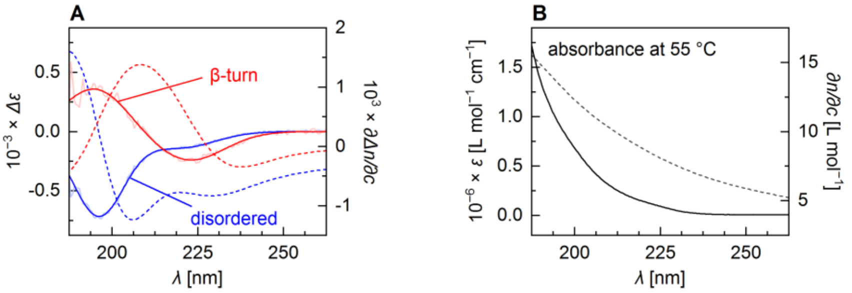

Figure S3. A: Gaussian fitted pure CD spectra (blue for disordered, red for β-turn) of $(VPGVG)_{40}$ and $\partial\Delta n(\lambda)/\partial c$ (dotted) from using equation S5. B: representative absorbance spectrum of $(VPGVG)_{40}$ (measured at 55 °C, solid) and the corresponding $\partial n(\lambda)/\partial c$ spectrum.

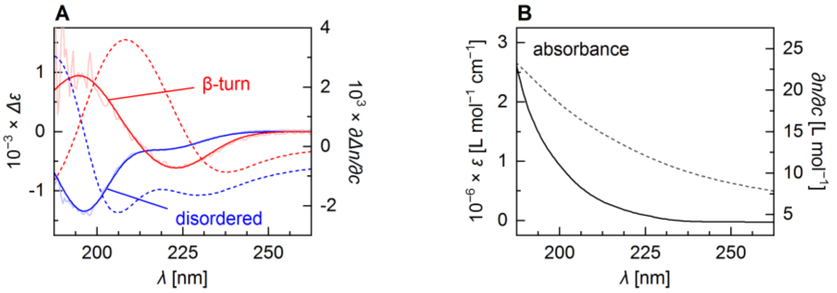

Figure S4. A: Gaussian fitted pure CD of $(VPGVG)_{60}$ and $\partial\Delta n(\lambda)/\partial c$ spectra (blue for disordered, red for β-turn, solid for CD, dotted for $\partial\Delta n(\lambda)/\partial c$). B: the spectra are the same as in Figure S3B, scaled by 1.5 to represent the absorptivity of $(VPGVG)_{60}$.

Confidence of matrix least-squares (MLS) global fitting

The fitting residuals $r(\lambda_i, T_j)$ as shown in Figure S5, Figure S6, and Figure S7 represent the difference between the raw data (non-weighted) and the fitting model given in equation (S1). The *R*-values given in the text are calculated by:

$$R = \frac{\sum_i \sum_j |r(\lambda_i, T_j)|}{\sum_i \sum_j |CD(\lambda_i, T_j)|} \tag{S6}$$

The *R*-values together with cosine similarity (cosθ) from the comparison of the pure species spectra obtained for disorderered and β-turn structure from the fitting of the $(VPGVG)_{40}$ and $(VPGVG)_{60}$, respectively, to the disorderered and β-turn structure spectra obtained for $(VPGVG)_{20}$ are summarized in Table S2.

Table S2. Confidence values for MLS global fitting.

Data set	*R*-value	cosθ disorderered	cosθ β-turn
$(VPGVG)_{20}$	0.09		
$(VPGVG)_{40}$	0.26	0.99	0.95
$(VPGVG)_{60}$	0.35	0.97	0.98

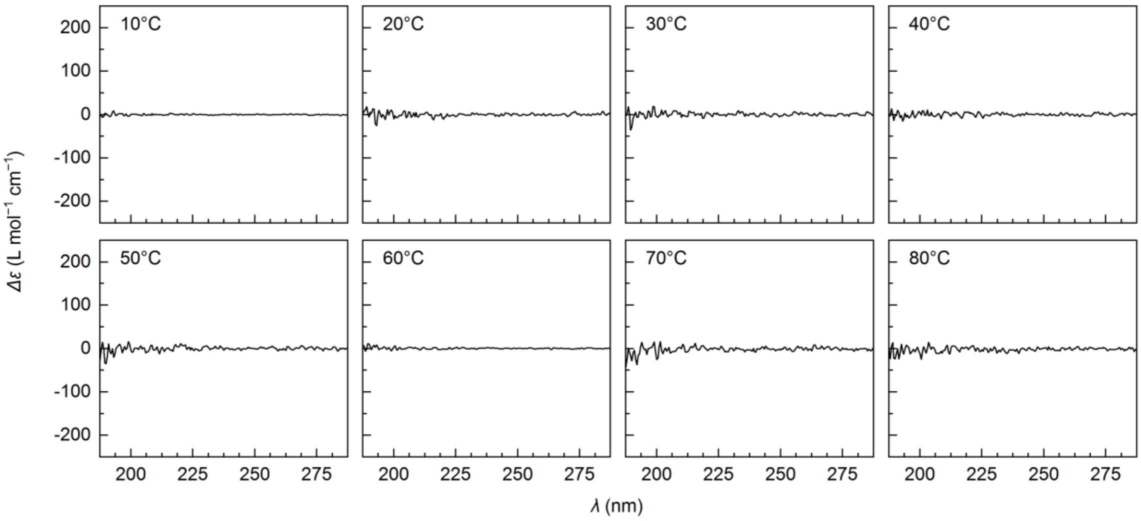

Figure S5. Fitting residuals for $(VPGVG)_{20}$. As no residual spectrum contains spectral features different from noise, the temperature-dependent process is perfectly described by the model.

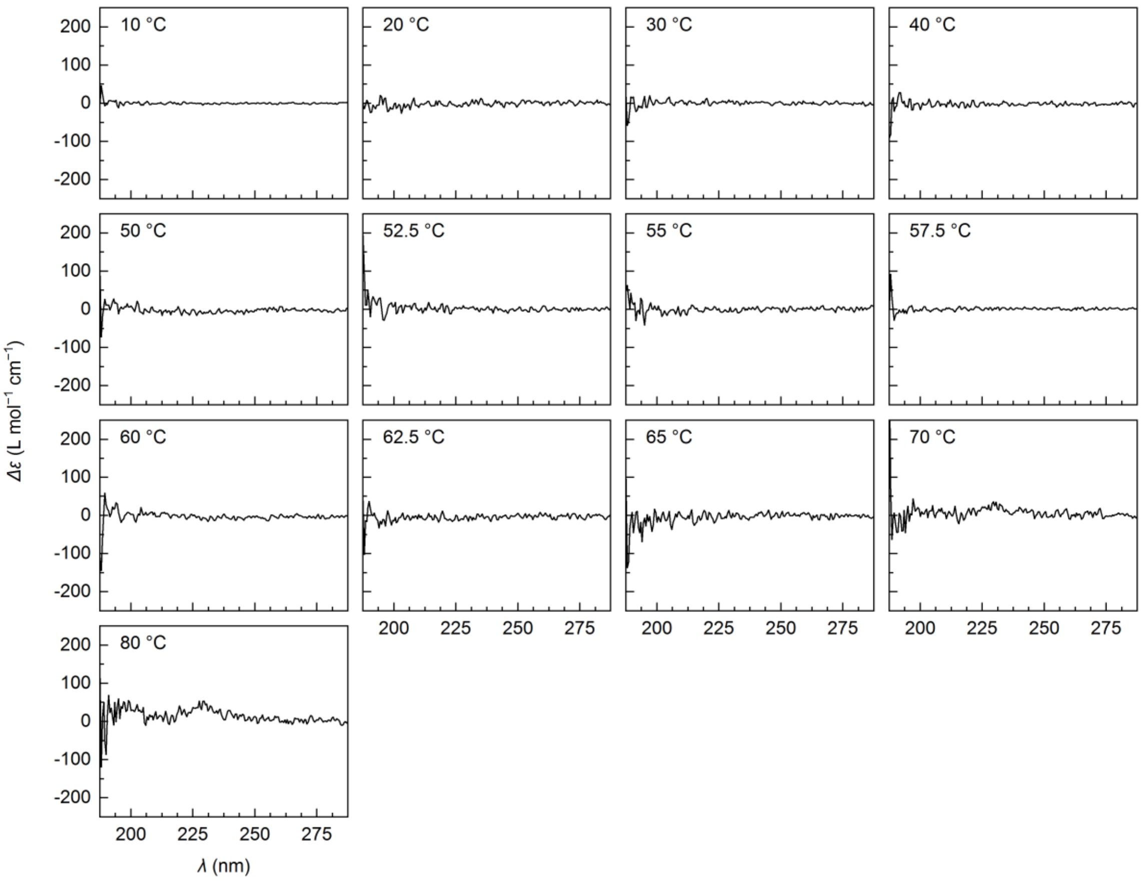

Figure S6. Fitting residuals for $(VPGVG)_{40}$. As expected, minor non-random features appear in the residuals above ITT (70 and 80 °C).

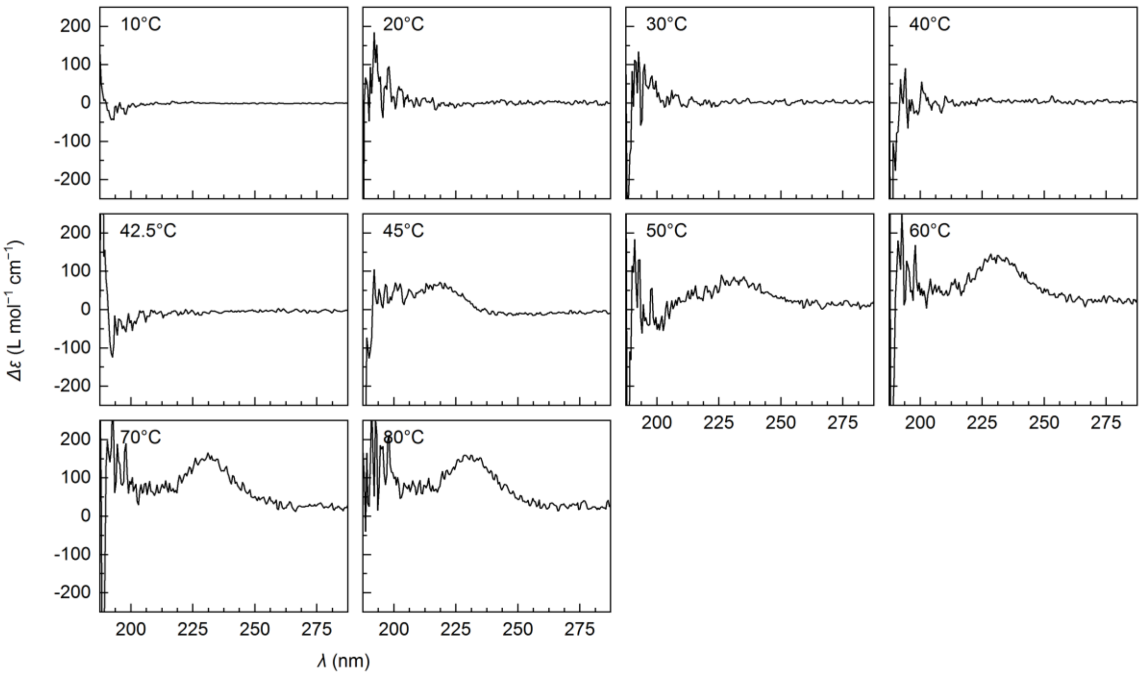

Figure S7. Fitting residuals for $(VPGVG)_{60}$. Due to the dramatic decrease of signal intensity above ITT, residuals calculated for spectra measured at 45, 50, 60, 70 and 80 °C clearly show non-random features.

Supplementary references

[40] E. Gasteiger, C. Hoogland, A. Gattiker, S. e. Duvaud, M. R. Wilkins, R. D. Appel, A. Bairoch, in *The Proteomics Protocols Handbook* (Ed.: J. M. Walker), Humana Press, Totowa, NJ, **2005**, pp. 571–607.

[41] V. A. Lórenz-Fonfría, H. Kandori, *J. Am. Chem. Soc.* **2009**, *131*, 5891–5901.

[42] R. W. Hendler, R. I. Shrager, *J. Biochem. Bioph. Meth.* **1994**, *28*, 1–33.

[43] H. Grubmüller, P. Tavan, *J. Chem. Phys.* **1994**, *101*, 5047–5057.

[44] a) C. Bustamante, I. Tinoco, M. F. Maestre, *Proc. Natl. Acad. Sci. U. S. A.* **1983**, *80*, 3568–3572; b) I. Tinoco, M. F. Maestre, C. Bustamante, *Trends Biochem. Sci.* **1983**, *8*, 41–44.

[45] V. Lucarini, K.-E. Peiponen, J. J. Saarinen, E. M. Vartiainen, *Kramers–Kronig Relations in Optical Materials Research*, Springer, Heidelberg, **2005**.
